# A longitudinal field study of commercial honey bees shows that non-native probiotics do not rescue antibiotic treatment, and are generally not beneficial

**DOI:** 10.1038/s41598-024-52118-z

**Published:** 2024-01-23

**Authors:** Kirk E. Anderson, Nathan O. Allen, Duan C. Copeland, Oliver L. Kortenkamp, Robert Erickson, Brendon M. Mott, Randy Oliver

**Affiliations:** 1 USDA-ARS Carl Hayden Bee Research Center, 2000 E. Allen Rd, Tucson, AZ 85719 USA; 2 Department of Entomology and Center for Insect Science, University of Arizona, Tucson, AZ 85721 USA; 3 ScientificBeekeeping.com, Grass Valley, CA USA

**Keywords:** Microbial ecology, Microbiome, Antimicrobials

## Abstract

Probiotics are widely used in agriculture including commercial beekeeping, but there is little evidence supporting their effectiveness. Antibiotic treatments can greatly distort the gut microbiome, reducing its protective abilities and facilitating the growth of antibiotic resistant pathogens. Commercial beekeepers regularly apply antibiotics to combat bacterial infections, often followed by an application of non-native probiotics advertised to ease the impact of antibiotic-induced gut dysbiosis. We tested whether probiotics affect the gut microbiome or disease prevalence, or rescue the negative effects of antibiotic induced gut dysbiosis. We found no difference in the gut microbiome or disease markers by probiotic application or antibiotic recovery associated with probiotic treatment. A colony-level application of the antibiotics oxytetracycline and tylosin produced an immediate decrease in gut microbiome size, and over the longer-term, very different and persistent dysbiotic effects on the composition and membership of the hindgut microbiome. Our results demonstrate the lack of probiotic effect or antibiotic rescue, detail the duration and character of dysbiotic states resulting from different antibiotics, and highlight the importance of the gut microbiome for honeybee health.

## Introduction

Honey bees (*Apis mellifera)* are commercially managed pollinators of major agricultural crops, contributing billions of dollars annually to the national economy^1^. As a social insect, the honey bee is host to many pathogens and parasites that reduce colony strength and survival^2,3^. Recent colony losses are attributed to larval disease and various pathogens, viral or bacterial in origin^4^. Thus, the proactive management of infectious and endemic disease is a major goal of beekeeping and agriculture. In the US and Canada, antibiotic application is widely used for treatment and control of two very different bacterial brood diseases; the highly virulent American foulbrood (AFB) and slower moving European foulbrood (EFB) disease. Following the recent microbiome revolution^5^, commercial probiotics have been used by beekeepers with the promise of reducing or controlling resident pathogens and restoring or improving the gut microbiome generally, or following antibiotic treatment^6^. Probiotic development and testing in honey bees has been limited^7–9^, and there is little empirical evidence supporting the effectiveness of non-native probiotics^10,11^.

If the introduced probiotics establish and propagate in the gut environment, they could affect colony health through the production of microbial metabolites or by excluding other microbes with less desirable function including pathogens and opportunists^10,12^. The honey bee worker has a resident (native) hindgut microbiome that is intimately tied to host metabolism, behavior and health^13–17^. It is highly structured, strain rich, taxonomically consistent, and alters subtly but predictably with age and behavioral role^18–22^. Recent taxonomic treatment show it is comprised of 10 genera from five phylotypes that consistently account for > 95% of the hindgut microbiota^19,23^.

Unlike vertebrate or fly models, the honeybee worker microbiome is highly predictable by species identity and very high values of species evenness, indicative of a healthy and highly co-evolved ecosystem wherein each species occupies a defined functional niche^14,24–26^. The gut microbiome is most dense in the hindgut where a bacterial biofilm runs from the pylorus to the rectum with the greatest concentration of bacterial cells found in the ileum and anterior rectum. The ileum is populated primarily by *Lactobacillus* spp., and two gram-negative bacteria; *Snodgrassella* and *Gilliamella*^14,26,27^*.* The rectum microbiome is primarily comprised of gram-positive lactic acid bacteria *Bombilactobacillus, Bifidobacterium* and a few major species of *Lactobacillus* that partition nutritional niche by strain and/or species^16,28^. Specialized host-microbial and inter-microbial relationships are unique to each gut section, and considered fundamental to host health^14,29^. Studies sequencing the gut as a whole typically result in libraries biased towards rectum species, but provide less resolution for the ileum biofilm, which often contains an order of magnitude fewer cells^30^.

The gut microbiota is disrupted by antibiotic application in the lab and field leading to substantial decreases in *Lactobacillius* and *Bifidobacterium* followed by dysbiosis and host susceptibility to disease^6,7,31^. In the United States, honey bees have been exposed to commercially available antibiotics for many decades^32^, and many members of the gut microbiome have acquired antibiotic resistance genes relative to those in honey bees with less recent exposure^33^. Following the application of antibiotics, some manufacturers recommend probiotic supplements for the purpose of improving the microbiome and general colony health (Table S1c,d). Probiotics are food or supplements that contain active or freeze dried microorganisms^34^, commonly lactic acid producing bacteria and yeasts including species of *Lactobacillus*, *Bacillus*, and *Saccharomyces*^35^. Popular honey bee probiotics are non-native and derived from environmental sources and from fermentation products often marketed as human probiotics. They are promoted with claims that they provide health benefits when consumed by controlling disease and improving or restoring the gut microbiome^36^. However, there is little evidence that non-native probiotics provide health benefits as claimed^10,11,37^.

In this contribution, we determine whether monthly application of either of two commercial and non-native probiotic formulations would influence pathogen loads or the gut microbiome when applied consistently over many months, or when applied following antibiotic treatment. We address three major questions; (1) Can the introduced probiotic microbes be detected in the worker gut? (2) Does monthly probiotic application affect the gut microbiome, fungal abundance or pathogen prevalence? and (3) Does the application of a probiotic accelerate microbiome recovery following antibiotic application? Because of this approach, we also detail the long-term effects of colony-level antibiotic treatment with both tylosin tartrate and oxytetracycline in a migratory beekeeping operation.

## Methods

### Colony metrics and management

We performed these experiments at two separate apiaries near Grass Valley, CA, USA, 38° 15′ 34″ N 122° 9′ 52″ W, at approximately 2900 feet elevation. We used three groups of 25 hives in a randomized block design with treatment and control groups equally divided between two apiaries (Tables 1 and S1). The experimental colonies were professionally managed, treated and sampled by scientificbeekeeping.com^37^. This commercial operation does not use antibiotics prophylactically, and none of the colonies had received antibiotic treatments previously, such that antibiotic resistance has not been selected for in this population. Prior to the experiment, colonies were roughly equalized to about 8–10-frame cluster size, and all colonies were headed by young queens. All colonies were visually screened for disease and sampled for *Varroa* mite infestation rate; those with counts above three mites per half cup of bees were treated with formic acid strips. Mites were subsequently controlled by applying oxalic acid/glycerin sponges to any colonies with mite counts above ten mites per half cup of bees. We performed two experiments using these colonies: “experiment one,” a long-term probiotic treatment, followed by “experiment two,” a rescue treatment where colonies were first treated with antibiotics, then with probiotics.

**Table1:** Colony sampling and treatment application timeline (2020–2021).

Date	Application/Sampling/Explanation	Sampling Time point
July 2^nd^, 2020	Inspect, score and randomly assign N = 76 colonies at two sites to one of three blinded probiotic treatments (A, B or Control; N ≥ 25 per treatment, N ≥ 12 per treatment per site)	
July—Nov. 2020	Apply probiotics as directed. (One tbsp. per colony per month of either commercial product (A or B) or powdered sugar (Control))	
Nov. 5^th^	Inspect and score hives to assess five months of probiotic treatment	
Dec. 2	Sample colonies following treatment with probiotics for five consecutive months. Samples also represent a baseline control for pre-antibiotic exposure. Assign colonies to antibiotic treatments (tylosin, oxytetracycline, or control)	Zero
Dec. 7	Apply antibiotics as directed. (Powdered sugar as control, see methods)	
Dec. 10	Apply antibiotics as directed	
Dec. 14	Apply antibiotics as directed	
Dec. 17	Sample colonies to characterize proximate effects of antibiotics, 10 days post antibiotic application	One
Dec. 19	Apply probiotics as directed (see methods)	
Dec. 26	Sample colonies to characterize probiotic assisted recovery, 19 days post antibiotic application	Two
Jan. 9	Sample colonies to characterize probiotic assisted recovery, 33 days post antibiotic application	Three

### Probiotic application

We applied two major commercial probiotics available on the market, here labeled A and B. The products are advertised to contain the same eight microorganisms with the exception of the fungus *Trichoderma reesei,* unique to Probiotic B. The eight microbes common to both probiotic formulations were *Lactobacillus acidophilus, Enterococcus faecium, Bifidobacterium bifidum, Lactobacillus plantarum, Bacillus subtilis, Bacillus licheniformis, Bacillus pumilus, and Saccharomyces cerevisiae* (Table S1c and d)*.* Both probiotic A and B are sold premixed with powdered sugar. To facilitate a double-blinded study, new containers were prepared for A, B, and C (powdered-sugar control). This ensured that neither the beekeepers, nor lab researchers knew which hives received each product or treatment. In experiment one, following manufacturer’s directions, hives were fed one tablespoon per month of A, B or C for five months (July 2020). Given the quantities advertised on the product, this results in the introduction of approximately 15 billion microbial cells per treatment. The experiment one probiotic trial spanned summer forage dearth and fall colony growth from supplemental feeding of pollen substitute. In experiment two, probiotic administration followed winter treatment with one of two different antibiotics. This period included both sporadic freezing temps and daytime temperatures that allowed flight activity (Table 1).

### Antibiotic application

To determine whether the probiotics facilitate the reestablishment of the worker gut microbiome following antibiotic treatment, we applied antibiotics when many commercial beekeepers apply either oxytetracycline or tylosin to control bacterial brood diseases, then applied probiotics afterward. In December, N = 54 colonies in probiotic groups A, B, and C were randomly assigned to receive either oxytetracycline or tylosin (Table S1a). Seven other colonies in probiotic group C were randomly assigned to receive powdered sugar (controls receiving neither probiotics nor antibiotics). Antibiotics were mixed with powdered sugar per manufacturer’s instructions (~ 200 mg active ingredient per 1 rounded tablespoon) and dusted over the cluster three times (Dec. 7, 10 and 14th, 2020). We sampled the hives for maximum antibiotic effect on Dec. 17th, applied probiotics on Dec. 19th, and then collected putative recovery samples at 7 and 21 days post probiotic application (Table 1).

Samples of ~ 50 bees were taken from every colony at each time point. Prior to sampling, we performed a fly-off assay, shaking bees from an outer brood frame into a plastic tub, allowing the older bees with flight training to fly off, leaving a sample of younger in-hive workers behind. From this subset of younger workers, we randomly collected approximately 50 bees into a 50 ml falcon tube. Bees were immediately frozen and shipped to the Carl Hayden Bee Research Center (CHBRC) on dry ice, then stored at − 80 °C pending molecular analyses.

### Pathogen detection and quantification

In experiment two, we determined whether probiotic application reduces pathogen levels by analyzing molecular markers of known disease at two time points after antibiotic application. The first sample (time one) was taken after antibiotic treatment and just prior to probiotic application to measure antibiotic effect on pathogen levels. The second sampling was 21 days after the application of probiotics, to determine probiotic effects on pathogen levels after antibiotic treatment (time three). These two time points capture the full effect of antibiotics and allow ample time for probiotic treatment to take effect (Table 1). To represent a taxonomic variety of known and prevalent pathogens, we analyzed the change in general fungal load, a bacterial pathogen of larvae (European foulbrood EFB, *Melissococcus plutonius*), a ubiquitous microsporidian (*Vairimorpha* (*Nosema*) *ceranae*), and four different viruses endemic to honey bees; deformed wing virus A and B, black queen cell virus, and chronic bee paralysis virus (Table 2). We first screened for EFB in workers using qualitative PCR and agarose gel electrophoresis, scoring bands that correspond to the predicted amplicon size as present/absent.

**Table 2:** Primers used to quantify pathogen levels.

Primer name	Primer sequence 5′–3′	Target	Citation
Actin-ref-F	TGCCAACACTGTCCTTTCTG	*Apis mellifera*	Amdam et al.^82^
Actin-ref-R	AGAATTGACCCACCAATCCA		
RPS18-ref-F	GATTCCCGATTGGTTTTTGA	*Apis mellifera*	Scharlaken et al.^83^
RPS18-ref-R	CCCAATAATGACGCAAACCT		
EFB-F_Evans	ACGCCTTAGAGATAAGGTTTC	*M. plutonius*	Evans^84^
EFB-R_Evans	GCTTAGCCTCGCGGTCTTGCGTC		
BactQuant-F	CCTACGGGDGGCWGCA	All Bacteria	Liu et al.^42^
BactQuant-R	GGACTACHVGGGTMTCTAATC		
FungiQuant-F	GGRAAACTCACCAGGTCCAG	All Fungi*	Liu et al.^43^
FungiQuant-R	GSWCTATCCCCAKCACGA		
Ncer-218-F	CGGCGACGATGTGATATGAAAATATTAA	*Vairimorpha ceranae*	Burgher-MacLellan et al.^85^
Ncer-218-R	CCCGGTCATTCTCAAACAAAAAACCG		
DWV-A-F	GAGATTGAAGCGCATGAACA	DWV type A	Teixeira et al.^86^
DWV-A-R	TGAATTCAGTGTCGCCCATA		
DWV-B-F	CTGTAGTTAAGCGGTTATTAGAA	DWV type B	Ryabov et al.^87^
DWV-B-R	GGTGCTTCTGGAATAGCGGAA		
BQCV-F	TGGTCAGCTCCCACTACCTTAAAC	black queen cell virus	Benjeddou et al.^88^
BQCV-R	GCAACAAGAAGAAACGTAAACCAC		
CBPvirus-F	GACCCCCGTTGGAACGACGC	chronic bee paralysis virus	Morimoto et al.^89^
CBPvirus-R	CGGACGACGATTGGCGCTCA		

*Primer set does not amplify Microsporidians (*Vairimorpha*, previously *Nosema*).

To increase the probability of molecular pathogen detection, we used a sampling and extraction protocol modified from Evans et al.^38^. Twenty-five whole bees per colony were homogenized in 12.5 ml RS lysis buffer (in pH 8.0 TE buffer: 0.27 M guanidine thiocyanate, 0.13 M ammonium thiocyanate, 0.03% Triton X-100, 286 mM β-mercaptoethanol) utilizing a rotor–stator homogenizer for 4 rounds of 30 s (120 s total) at 11,000 RPM. For each homogenate, a 1.8 ml subsample was immediately collected and frozen on dry ice, then stored at − 80 °C until extraction with a modified GeneJET RNA Purification kit protocol (ThermoFisher #K0732). Prior to RNA extraction, homogenates were thawed at 60 °C for 4 min, vortexed at max speed for 60 s, then 300 μl of each homogenate was added to a 2 ml bead-beating tube containing 100 ul of 0.1 mm silica-zirconia beads and 300 μl kit lysis buffer. Samples were bead-beaten for 30 s, then received 10 μl Proteinase K and 290 μl TE buffer and incubated at room temperature for 10 min. RNA was further purified following manufacturer’s protocol for Insect Total RNA.

#### Real-time qPCR

We generated template cDNA from the pooled colony sample as follows: 8 μL of extracted RNA was treated with DNase I (1 μL enzyme + 1 μL buffer, Ambion #AM2224), then the whole 10 μL reaction was used as template in a 20 μL cDNA synthesis per manufacturer’s protocol (RevertAid First Strand cDNA Synthesis Kit, ThermoScientific #K1622). The 20 μL cDNA product was diluted with 180 μL of nuclease-free water prior to qPCR.

The cycling conditions for qPCR were; 95 °C for 5 min, then 45 cycles with 94 °C for 20 s and 60 °C for 30 s, followed by a high-resolution melt curve. Reactions utilized Luna Universal qPCR Master Mix (NEB #M3003E) in triplicate on a CFX96 Real-Time PCR Detection System (Bio-Rad). Each 12 μL reaction contained 6 μL Luna mix, 0.5 μL of forward and reverse primers (10 μM), 2 μL of cDNA template, and 3 μL H2O. To confirm the absence of contaminant DNA and primer dimers, no-template controls (consisting of reaction mix and water) and melt-curve analyses were included on each qPCR plate. Relative microbial abundance was estimated via 2^-ΔΔCq^39^ method using two honey bee mRNA reference genes for normalization (Actin and RPS18), and all values were then expressed relative to mean microbial load in the Time Point 1 control hives. These normalized data were log10 transformed and evaluated using one-way ANOVA with Tukey’s HSD post-hoc test. All analyses were conducted in JMP_ v16.2.0 (JMP_1989–2007) and/or SAS_v9.4^40^.

### *Vairimorpha* (*Nosema*) quantification

For time points zero through four (Table 1) *Vairimorpha* (*Nosema*) abundance was also quantified by counting spores under magnification from a pooled colony sample^41^. Briefly, the microscopic counting of *Vairimorpha* spores was according to standard methods^41^ with minor modifications. Bee tissue homogenates were generated by blending 25 whole bees per sample in 25 ml of distilled water using a Rotostat @ ~ 11 k RPM for 2 s. We allowed the homogenate to settle for one minute to separate solution from the bee exoskeleton and other recalcitrant tissues. We then collected 1 ml subsample of solution containing spores and pipetted onto the hemocytometer. We allowed 120 s for the solution to settle in preparation for microscopy and counting. Five squares at random were counted at 400× magnification on both sides of the hemocytometer to produce an average undiluted spore count per bee. If all five squares contained < 30 spores total, we counted all 25 squares of the hemocytometer to estimate *Vairimorpha* spore loads.

### Microbiome size and structure

We performed high-throughput sequencing of 16S rRNA bacterial gene to determine the microbiome structure of individual hindguts (n = 240). We dissected hindguts into 2-ml bead-beating tube containing 0.2 g of 0.1-mm silica beads and 300 μl of 1X TE buffer. Samples were bead beaten and homogenized for 2 min at 30-s intervals. To each sample, 100 μl lysis buffer (20 mM Tris–HCl, 2 mM EDTA, 5% Triton X-100, 80 mg/ml lysozyme, pH 8.0) was added followed by incubation at 37 °C for 30 min. DNA was then purified using a Thermo Fisher Scientific GeneJet Genomic DNA Purification Kit according to the manufacturer’s instructions for gram-positive bacteria. Sequencing was performed at the University of Arizona Genetics Core (UAGC) on a MiSeq (v3 PE-300 kit) following the manufacturer’s DNA library preparation protocol for amplification of the V3–V4 region of the 16S rRNA gene.

We estimated bacterial and fungal load in individual worker gut segments (midgut and hindgut) using degenerate bacterial primers and qPCR calibrated with a dilution series of plasmid standards^42^. We created plasmid vectors using Invitrogen’s pCR2.1 TOPO cloning vectors per the manufacture’s specifications. The 16 s gene template was amplified using BactQuant forward primer 27F (5′-AGAGTTTGATCCCTCAG-3′) and reverse prime 1522R (5′-AAGGAGGTGATCCAGCCGCA-3′)^42^. The 18 s gene template was amplified with forward primer PanFungal_18S_F (5′-GGRAAACTCACCAGGTCCAG-3′) and reverse primer PanFungal_18S_R (5′-GSWCTATCCCCAKCACGA-3′)^43^.

### Bioinformatics

16S rRNA gene sequences were processed using MOTHUR v.1.44.3^44^.Briefly, paired end reads were joined using the make.contigs command. After the reads were joined, we removed the first and last five nucleotides using the SED command in UNIX. Sequences were screened to remove ambiguous bases, using the screen.seqs command. Unique sequences were generated using the unique.seqs command. A count file containing group information was generated using the count.seqs command. Sequences were aligned to BEExact^45^ database using the align.seqs command. Sequences were filtered to remove overhangs and gaps using filter.seqs. The unique.seqs command was run again to remove new redundancies from filtering. A precluster step using pre.cluster was performed. Chimeras were removed using chimera.uchime command^46^. Sequences that were not bacterial in origin were removed using the remove.seqs command. All unique sequences with only one or two reads (single/doubletons) were removed using the AWK command in UNIX. A distance matrix was constructed for the aligned sequences using the dist.seqs command. Unique OTUs were merged at the species-level with the merge.otus command. The summary.single command was used to generate the number of observed OTUs (sobs), Shannon diversity, and Shannon evenness diversity metrics by treatment and time.

### Statistical analysis

The 16 most abundant species designations were normalized by absolute abundance by first calculating the relative amount of each species based on raw read count per sample. Absolute species abundance was calculated as the product of species relative abundance and 16S rRNA gene copies determined with BactQuant. Next, we assigned 16S rRNA gene copy number to each species based on their closest taxonomic representative^47^. A sum of species 17–229 was normalized with 4.2 gene copies, the mean 16S rRNA gene copy number averaged across all known bacteria^48^. Next, the data were center-log ratio (CLR)-transformed using the software CoDaPack^49^. CLR normalization is necessary to account for the compositional nature of 16 s amplicon sequence data, which makes standard statistical methods inapplicable before transformation. CLR-normalized data were used to investigate changes in microbiota structure using multivariate analysis of variance (MANOVA), examining probiotics and antibiotics as independent variables and 17 dependent variables that represent the honey bee gut microbiome. Pillai’s Trace test statistic was used for all MANOVAs to account for deviations in normality and homogeneity of covariance. Because MANOVAs compare only relative abundance changes in the microbiota as a whole, we also used Wilcoxon tests on the qPCR-normalized bacterial abundances by treatment to examine absolute abundance changes in individual species without regard to overall microbiota structure. We report the false discovery rate (FDR) and apply a Bonferroni correction to account for multiple comparisons. We controlled for both antibiotic and probiotic application in separate models. Principle component analysis (PCA) was performed on CLR-adjusted scores plotting the relationship of bacterial community composition with probiotic and antibiotic treatments. Alpha diversity metrics were compared by time point and treatment with two-way ANOVA followed by Tukey’s HSD post-hoc.

## Results

### 16S rRNA gene sequencing

Following six months of prophylactic treatment with probiotics, the abundance and prevalence of pathogens did not differ between controls and either probiotic treatment (Fig S1, Table S2a,b). The application of probiotics as a hypothesized recovery from antibiotics revealed no difference in abundance or prevalence of tested pathogens from time one to time three (Table S2a). Pathogen levels in antibiotic treated colonies were not significantly different from controls, except for black queen cell virus (BQCV), which was more abundant in colonies treated with antibiotics, but we note the small sample size of control colonies used for this comparison.

Considering both time points, only 18% (16 of 91) of EFB samples were positive as determined by presence /absence of an amplicon in agarose gel corresponding to the expected base pair size. Because the prevalence of *M. plutonius* was exceedingly low, and unassociated with probiotic or antibiotic treatment, we did not analyze *M. plutonius* with qPCR.

We analyzed four different viruses by qPCR, none of which differed in prevalence or abundance by probiotic treatment (Table S2a,b). The chronic bee paralysis virus (CBPV) was detected in 90% (82 of 91) of samples, the black queen cell virus was detected in 91% (83 of 91) of samples, deformed wing virus A and B were detected in 93% and 100% of samples respectively. While probiotic treatment was represented by a robust sample size, the control colony sample sizes for antibiotic treatment were too small to rely on statistical tests with any confidence. However, colonies treated with the antibiotic tylosin differed for CBPV abundance when comparing time one and time three (*p* = 0.009) and BQCV was more abundant in colonies treated with antibiotics (*p* < 0.001).

We determined the abundance of fungi in individual workers using universal fungal primers. At time zero, following six months of prophylactic probiotic treatment, individuals from colonies treated with Probiotic A contained a greater abundance of fungi in their hindguts as compared to those from control colonies (F_2,57_ = 3.9, *p* = 0.03). None of the other time points or tissues differed for fungal abundance by probiotic application. We also quantified the relative abundance of fungi in pooled worker samples by colony at time one and three, and again found no differences by probiotic treatment (Table S2a).

We determined the change in *Vairimorpha (Nosema*) abundance with two methods. The molecular method (qPCR) revealed no differences from time one to time three, and generally low overall *Vairimorpha* abundance relative to host gene expression. The microscopy method revealed no difference in absolute spore abundance across the four time points, nor between treatment groups. (Table S2e).

### Microbiome details

Next generation sequencing of the 16S rRNA gene returned 14,031,800 raw reads (Table S3a). Following quality review, we removed seven failed libraries, and retained 14,023,569 total reads across 233 libraries. This curated data set had a mean of 60 k reads per library, with values ranging from 12 to 102 k. The median value per gut library was 59 k, and only three of 233 analyzed libraries contained less than 30 k reads. Following the merger at species level, we distilled 229 bacterial species from a raw total of 11, 700 unique OTU’s (Table S3b). For the MANOVA and Wilcoxon rank sum analyses, we chose to analyze the top 16 species accounting for 95% of the read total. We grouped the remaining 5% of the reads (species 17–229) in the MANOVA analysis as a measure of diversity abundance. Not to be confused with established diversity metrics or a species-specific OTU, this value represents the relative abundance and summation of the rare species with very low or sporadic abundance in the worker gut^18^.

### Probiotic treatment

Variation in the gut microbiota explained by probiotics was insignificant regardless of the analyzed time, type of analysis, size or inclusiveness of the model (Table S4a,b). In the first experiment, the microbiota of colonies treated with either probiotic for six months did not differ from that of control colonies (Fig. S1). The full MANOVA model investigating CLR scores revealed no overall probiotic effect (Pillai's Trace = 0.18, F_32, 84_ = 1.12, *p* = 0.33). Similarly, Wilcoxon analysis performed for each individual OTU revealed no differences between the controls and probiotic A or B following Bonferroni correction for multiple comparison (Table S4a).

In experiment two, the application of probiotics did not hasten recovery of the gut microbiome following antibiotic exposure. The worker gut microbiota associated with colonies receiving probiotics did not differ from that of colonies receiving sugar-only controls at any time (Pillai's Trace = 0.18, F_32, 382_ = 1.17, *p* = 0.25). Wilcoxon analyses were again consistent with MANOVA analysis, revealing no difference between control colonies and those treated with probiotics (Table S4a,b).

To determine whether the probiotic species establish or persist in the gut, we searched our sequencing results internally for the occurrence of the nominal probiotic species (Table S3a). Despite deep sequencing with high-throughput technology, three of the seven introduced probiotic bacterial species were not detected in the worker gut; *Lactobacillus acidophilus, Bacillus subtilis* and *Bacillus pumilus*. We detected four bacterial species that correspond to the nominal introduced probiotics: *Enterococcus faecium, Lactobacillus (Lactiplantibacillus) plantarum, Bifidobacterium bifidum* and *Bacillus licheniformis* occurred with negligible prevalence and abundance (Table S3a). The latter two were each represented by a single unique sequence, while *L. plantarum* and *E. faecium* were represented by two and three unique sequences respectively. We used a test of proportions to compare patterns of probiotic species occurrence comparing controls to probiotic. At time two, the sampling event with greatest temporal proximity to probiotic application, the prevalence of nominal probiotic species in the worker gut was significantly associated with probiotic treatment (z = 3.6, *p* < 0.0001).

### Antibiotic treatment

**Figure 1:**
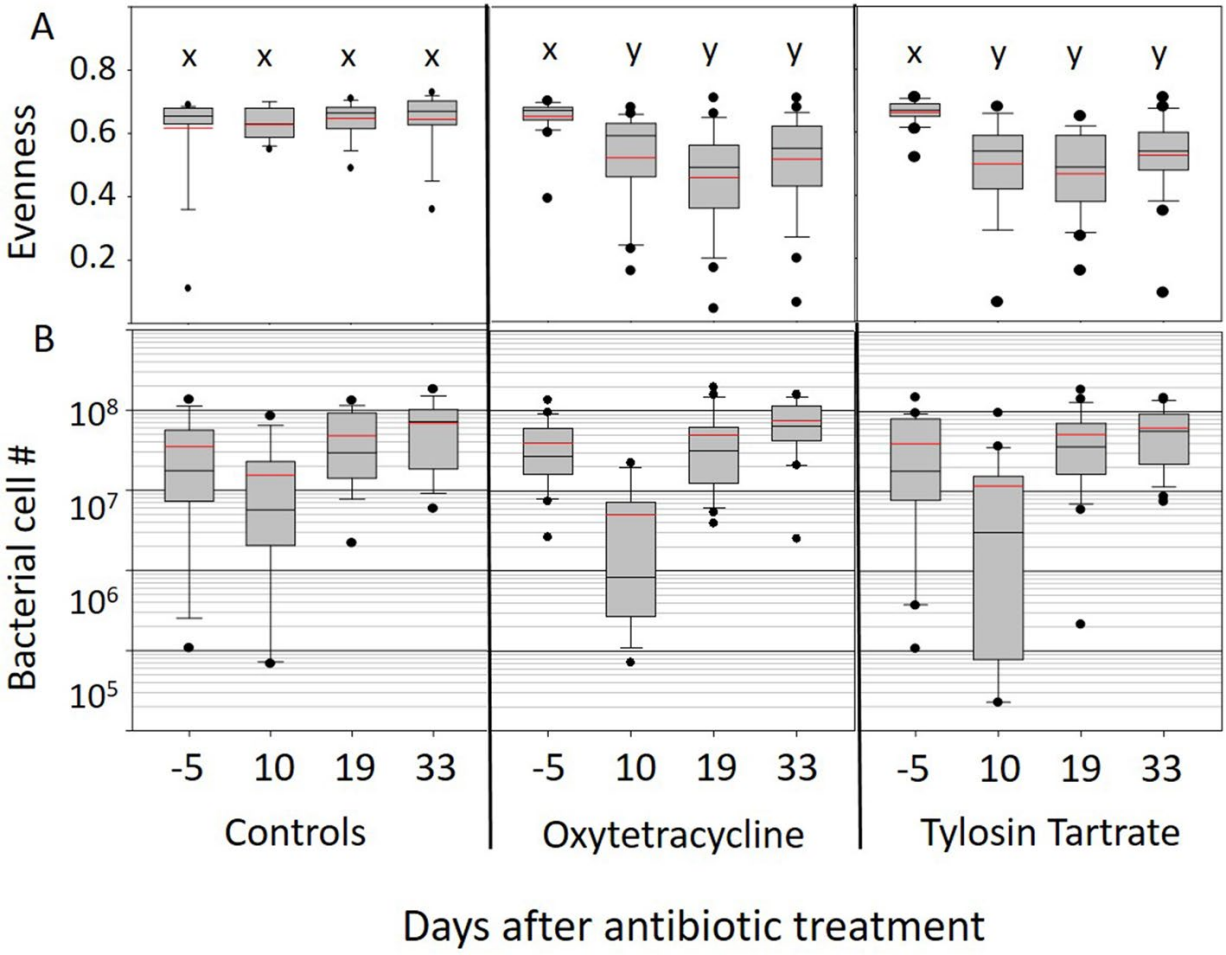
Two metrics that illustrate changes in the gut microbiome over time associated with antibiotic treatment. The top panels display Shannon Evenness, a metric of gut microbial diversity with values ranging from complete species dominance (0.0) to absolute evenness (1.0). Lower panels show microbiome size, determined by BactQuant, and expressed as total bacterial cell number. The x-axis lists four time periods sampling control colonies and colonies treated with either oxytetracycline or tylosin tartrate; five days before treatment, ten days after treatment, nineteen days after treatment, and thirty-three days after treatment. Grey boxes contain 50% of the variation, red line is the mean and black the median, whiskers are at 10 and 90% and dots are outliers. Within each panel, box plots with the same letter do not differ (*p* < 0.05).

**Figure 2:**
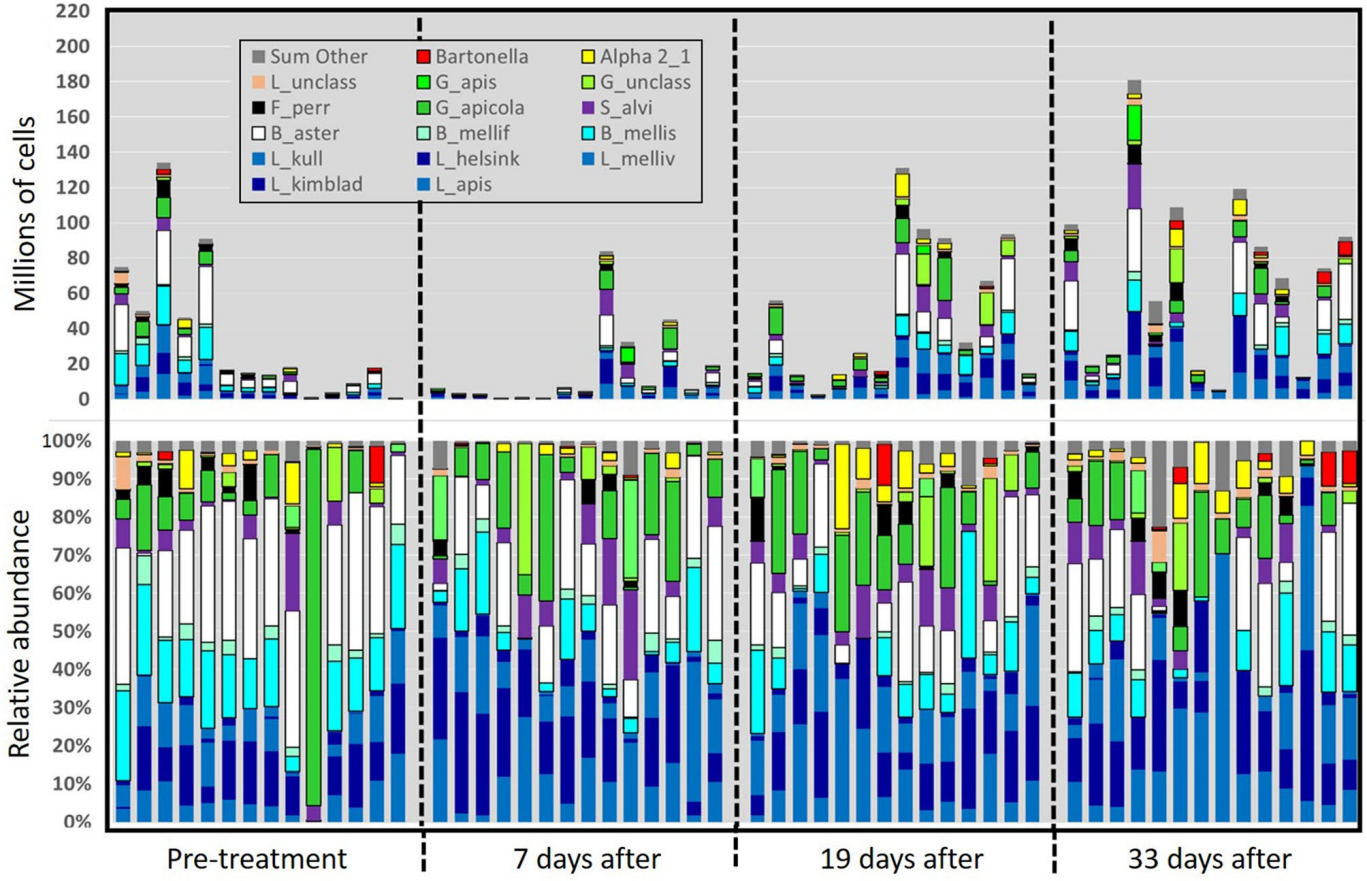
Gut microbiomes of workers from control colonies, sampled at four time points during the winter of 2020–2021. The bottom panel represents relative abundance with percent of total listed on the y-axis; the top panel is BactQuant normalized (absolute) abundance, with values listed on the y-axis representing millions of cells (× 10^6^). The x-axis refers to the timing of antibiotic application in the treatment colonies (see also Figs. 3 and 4).

**Figure 3:**
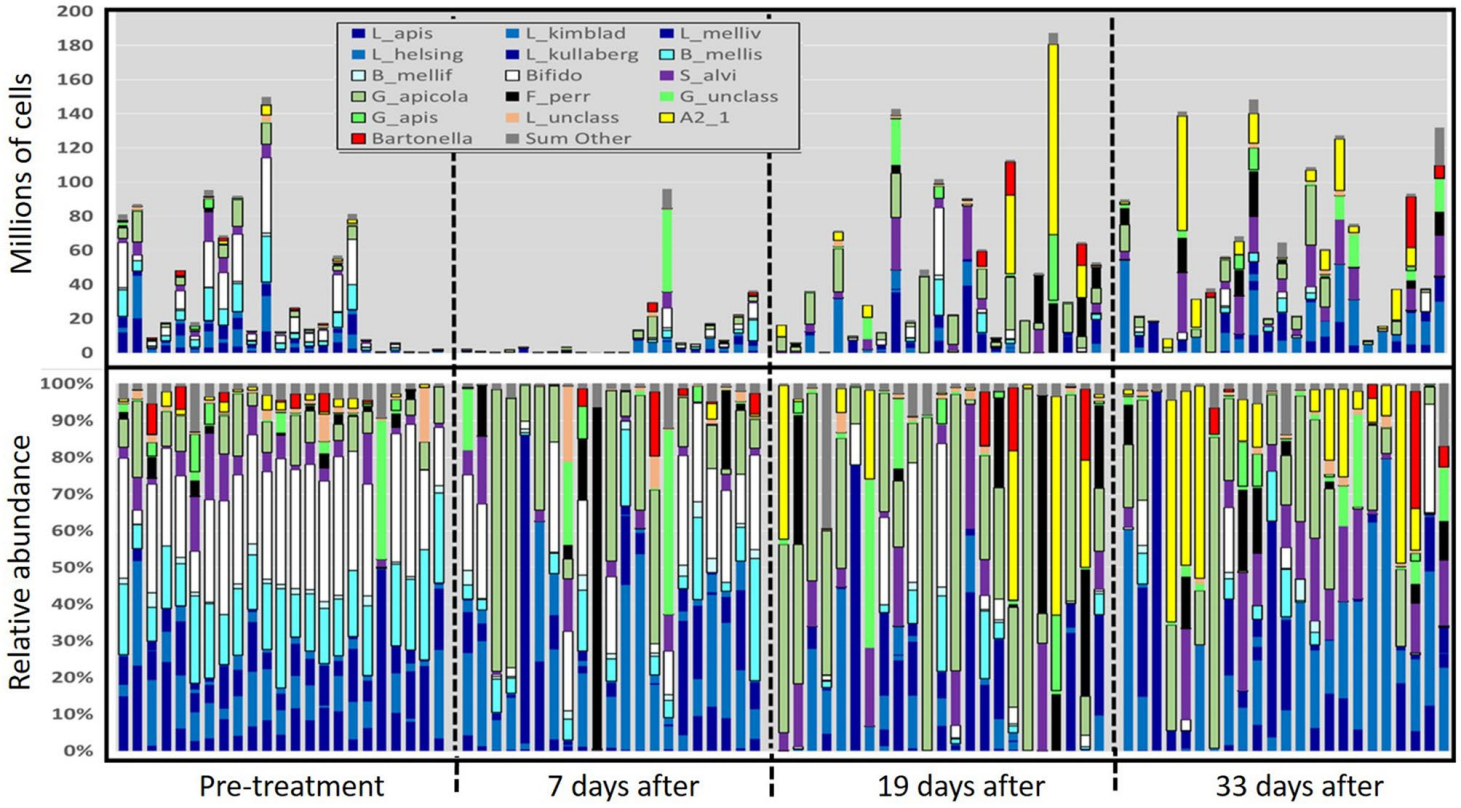
Gut microbiomes of workers from colonies treated with oxytetracycline, sampled at four time points during the winter of 2020–2021. The bottom panel represents relative abundance with percent of total listed on the y-axis; the top panel is BactQuant normalized (absolute) abundance, with values listed on the y-axis representing millions of cells (× 10^6^).

**Figure 4:**
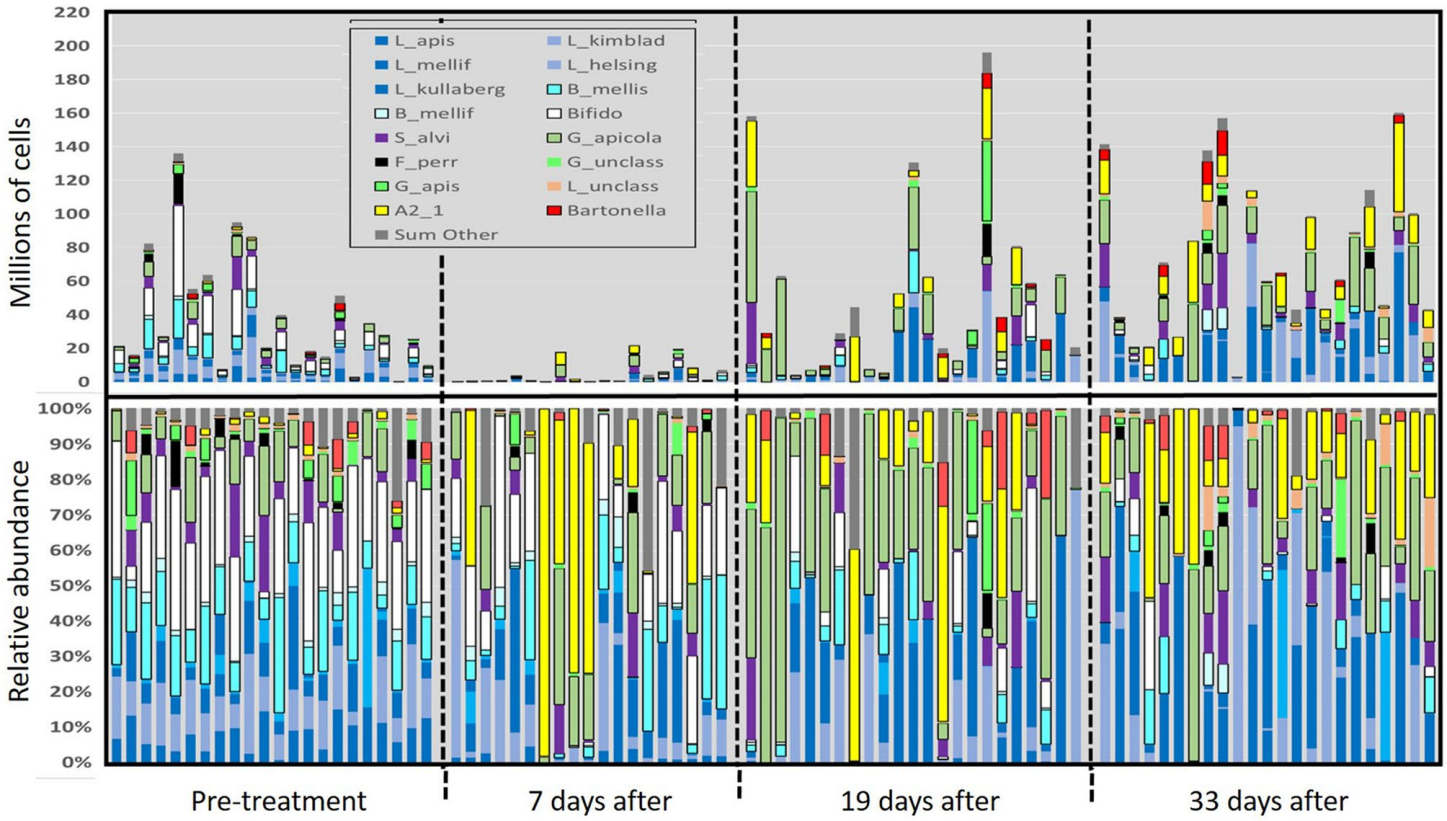
Gut microbiomes of workers from colonies treated with tylosin tartrate, sampled at four time points during the winter of 2020–2021. The bottom panel represents relative abundance with percent of total listed on the y-axis; the top panel is BactQuant normalized (absolute) abundance, with values listed on the y-axis representing millions of cells (× 10^6^).

Due to the sparsity of bacterial sequences in the gut corresponding to the introduced probiotic, and lack of microbiome variation that could be attributed to probiotic treatment, our downstream analyses considered the variation attributable to antibiotic treatment without accounting for probiotic in the model. Variation in the gut microbiota due to antibiotic differed significantly by time, and very different microbiotas resulted from treatment with tylosin vs. oxytetracycline (Figs. 1, 2, 3, 4). At time one, directly following the three-day antibiotic application, microbiome size was reduced significantly in both treatments. Following the pattern of decreased microbiome size directly following antibiotic treatment, six amplicon libraries from time point one failed due to insufficient amounts of microbial DNA present in the reaction or gut. These six libraries averaged 5 × 10^3^ gene copies according to qPCR, insufficient to produce a reliable 16S rRNA gene amplicon library. Two of these failed libraries were treated with oxytetracycline (sample numbers 71 and 92) and four with tylosin (76, 86, 88 and 96). One of the blank Illumina reactions used as a negative control confirm the contaminant OTU’s amplified in the much smaller time one libraries (Table S3b).

The structure of the microbiota differed by both time (Pillai's Trace = 0.36, F approx. = 2.85, *p* < 0.0001) and type of antibiotic (Pillai's Trace = 0.69, F approx. = 3.91, *p* < 0.0001). Following FDR correction, eleven of the 17 analyzed taxonomic categories differed by antibiotic, and two by time with no interaction effect (Table S5a-h). Following an initial decrease in both treated and control colonies at ten days post treatment, we saw microbiome size rebound above time point zero levels at time points two and three. With both antibiotics, gram-positive species *Bifidobacterium* a*steroides* and *Bombilactobacillus* (both *B. mellifer and B. mellis*) were depleted progressively over the assessed period (Figs. 1, 2, 3, 4). The immediate effect of antibiotics at 10 days post-application was characterized by the loss of bacterial mass and a shift in relative abundance favoring *Gilliamella apicola* and phylotype Alpha 2.1 in tylosin treatments, and *Gilliamella* spp. in the oxytetracycline treatments (Figs. 2, 3, 4). At time points two and three post antibiotic application, depletions of gram positive bacteria were replaced by either *Gilliamella apicola*, *Gilliamella* spp., or Alpha 2.1, sister group to *Commensalibacter* (Figs. 2, 3, 4).

### Alpha diversity

To test the hypothesis that probiotic application aids recovery of the gut microbiome, we examined effective number of species, Shannon diversity, Shannon evenness, and observed species number. All metrics differed significantly by antibiotic treatment but probiotic application had no effect on microbiome recovery (Table S4a,b). In the controls, all four metrics remained high and invariable across the four time points regardless of the change in microbiome size. At time one (10 days post antibiotic treatment), all metrics decreased significantly in response to oxytetracycline treatment, and following correction for multiple comparisons. Treatment with tylosin produced a similar trend, and all measures but diversity remained significant after FDR correction (Table S5d). At time two (19 days), the effect of both antibiotics was greatly amplified, and all four metrics were reduced significantly. Diversity measures increased at time three (33 days) but still remained significantly lower compared to the controls (Table S5f). Not a single gut microbiome at 33 days post antibiotic application resembled the even structure or taxonomic representation of the sham-treated controls or pre-antibiotic samples (Table S5f).

## Discussion

Here we show that non-native probiotics marketed as a beneficial medicine for honey bees have no effect on pathogen prevalence or the honey bee gut microbiome. Our double blind test of probiotic effectiveness examined a large cohort of commercial colonies under two distinct circumstances; (1) following prophylactic probiotic application every month for seven months, and (2) the application of probiotics following antibiotic induced gut dysbiosis. In total, we assayed seven common pathogens, but found no meaningful differences in pathogen abundance or prevalence associated with probiotic application. High throughput DNA sequencing resulted in 14 million DNA sequences representing the hindgut microbiomes of 233 workers, but we detected only a scattered and sparse representation of microbes that could be attributed to probiotic application. Consistent with our molecular results, a colony-level analysis of this same sample set revealed no effect of probiotic treatment on colony weight or number of worker bees^37^. We conclude that the introduced microbes have no effect upon the host organism, primarily because they could not survive or effectively propagate in the colony or gut environment.

The introduced probiotics are not native to the honey bee, hive or the floral environment, but have a long history of use in humans and farm animals^50,51^. Both probiotics contain seven of the same bacterial species in the same concentrations, all generally regarded as safe, and authorized for use in fermented foods and consumption by humans and large animal agriculture. The probiotics are advertised to produce many effects, some of which may not rely on establishment in the gut (Table S1c-d). Observations in the field indicate that the probiotics were readily consumed by worker bees. Frequent trophallaxis among worker bees allows the probiotic microbes access to both worker guts and colony resource space^52^. Despite this level of exposure and a mean of 60,000 DNA sequences per gut, we detected only four of the nominal bacterial species in the gut, all occurring with negligible abundance and prevalence at one week post probiotic treatment. The commercial probiotic formulations were applied as directed, but we did not independently verify the listed counts for colony forming units, advertised as > 10^9^ per gram by the manufacturer.

Based on the most recent and complete taxonomy^45^, none of the introduced probiotic species are native to the honey bee gut or social resource space with the possible exception of *Enterococcus faecium*^36^. Taxonomic assignments resulting from earlier and largely non-curated databases named various lactic acid bacteria that correspond to the introduced probiotic species including *Lactobacillus plantarum, L. acidophilis*, and *Bifidobacterium bifidum.* However, updated reference sets associated with new bioinformatic tools, and the use of amplicon sequence variants demonstrate that this nomenclature was a consequence of taxonomic limitation and clustering OTUs at 97% similarity in earlier publications, and these species do not belong to the honey bee microbiota^45^.

The ecology of the introduced probiotics might suggest that some of the species could survive for a short period following colony introduction. These characteristics include sporulation (*Bacillus* spp.) and tolerance for temperature extremes and acidic environments. For example, *L. acidophilus* achieves its highest growth rates in slightly acidic media of pH 5.5–6.0, but ceases growth below pH 4.0^53^. Honey and the worker foregut (social stomach or crop) are typically around pH 4.0 presenting a barrier to probiotic establishment. Similarly, *L. acidophilus* is among the least oxygen tolerant lactobacilli^54,55^ which is likely a disadvantage throughout the oxygen rich social resource space of the colony. In addition to these abiotic factors, a number of native and host co-evolved *Lactobacillus* species reside in the honey bee worker hindgut, representing a competitive barrier to establishment. Given the nature of the honey bee and its built environment, we conclude that the vast majority of non-native probiotics are unlikely to survive or reproduce in numbers required to influence colony health. However, probiotics formulated with native honey bee gut microbes may be successful^56^. The process of introduction is greatly enhanced when applying probiotic species that are adapted to the honey bee gut and colony, and bees treated with native probiotics experience reduced negative effects of antibiotic exposure^10,21,57^.

The honey bee hosts highly co-evolved microbiomes that occur in the gut, and throughout the social resource space of the colony, a niche best defined by its association with social nutrient processing. Because the honey bee constructs and maintains a built structure to produce young and store food, this broadly defined niche includes mouthparts and midguts of both queens and workers, stored and secreted food, developing larvae, the social glands and social stomach^9,58,59^. The niche is generally aerobic or microaerophilic supporting a rich native microbiota comprised of extremophilic bacteria and yeasts^60,61^. It is highly antibacterial due to the addition of host-supplied enzymes, microbial enzymes, and hygroscopic and acidic properties of honey^62^. Unlike the hindgut, which is typified by species evenness, these niches are characterized by species dominance. Their highly antibacterial character creates scramble competition among the most fit strains of extremophiles^63,64^. In contrast, the worker hindgut tissues are highly competitive microbial niches and generally anaerobic, containing the greatest density of microbial cells^14,17^. Many years of data have shown conclusively that exogenous (non-native) microbes are not tolerated in the worker gut or throughout social resource space^10,61^.

### Antibiotic effects

Consistent with previous work^7,57^, we confirmed that both beekeeper-applied antibiotics produce dysbiotic effects on the gut microbiome. However, our sampling revealed that worker gut dysbiosis endured in colonies for greater than one month following initial exposure, a poorly known phenomenon^57,65,66^. We found that each popular antibiotic produces a unique dysbiotic signature, but there are some similarities. In general, both antibiotic treatments generated an environment of opportunism for non-susceptible and native species including those known to possess antibiotic resistance genes^33^. Following treatment with either antibiotic, rectum endemics *Bifidobacterium* and *Bombilactobacillus* were depleted progressively as *Gilliamella apicola* experienced competitive release becoming the dominant species in many samples. *Gilliamella* shares many metabolic capabilities with *Bifidobacterium* including variable carbohydrate metabolism^13,17,67,68^. *Gilliamella apicola* inhabits the ileum of healthy samples so its increase in this case may indicate niche expansion into the rectum due to a loss of metabolic competition. Alternatively, the midgut can harbor 10^8^ *Gilliamella* overwinter^69,70^ and our assessment of midgut microbiome size is positively correlated with *Gilliamella* abundance in the gut. Similar to previous results^7,57,71^ our data set reflects a reduction of abundance and diversity within many species clusters, indicative of broad spectrum effects.

We repeatedly sampled the same colonies at four time points overwinter to capture the full effect of antibiotics and probiotics. At time one, the control colonies varied markedly for microbiome size and fungal load concomitantly with those receiving antibiotic treatment. Inter-colony transmission of antibiotics may have affected our results. Although controlled in part by supplying sugar solution to fuel colony thermoregulation, this may suggest an apiary effect due to drifting or robbing bees (those moving between colonies) and honey contamination across colonies^66^. These results may also reflect local weather variation (https://www.timeanddate.com/) and dietary changes throughout the winter.

It is well demonstrated that the highly predictable structure of the honey bee gut microbiome improves host health^14^, and the control colonies remained stable for community evenness and other diversity metrics throughout the winter. Alterations from this even structure are associated with disease progression and greater mortality^6,29,72^. Some of the core gut species alter significantly with task, age and overwintering^18,29,73^, but it is mostly unknown how the microbiota behaves overwinter^69,73^ or how various forms of dysbiosis or alternate gut enterotypes affect honey bee health^74,75^. Perhaps replacement by *Gilliamella apicola* and *Commensilabacter* (Alpha 2.1) only represents a slightly substandard microbiome although antibiotics have been shown to increase pathogen susceptibility by disrupting the gut microbiome and associated immune physiology^6,7,76^. When sequenced or analyzed as a whole, the healthy honey bee gut microbiome is represented by an even distribution of species^77^. If evenness represents a healthy gut microbiome or ecosystem, it follows that increasing species dominance represents a dysbiotic microbiome^75^. Despite what may represent environmental variation across our time points, all three major diversity metrics, including evenness, remained high and invariable in control colonies across the four winter dearth time points consistent with a previous colony-level study^57^.

The prevalence and abundance of Alpha 2.1 increased dramatically after antibiotic treatment. Sister group to genus *Commensalibacter*, Alpha 2.1 is associated with queen youth and fecundity^58,78,79^. Alpha 2.1 can increase in absolute abundance with worker age so our increases in Alpha 2.1 may represent a sampling of progressively older bees throughout the winter, however in similar winter conditions workers show low mean longevity in December^69,80^. How Alpha 2.1 functions as part of the worker microbiome is unknown, but it seemed to replace groups such as *Bifidobacterium,* associated with the fermentation of polysaccharides found in pollen. Alpha 2.1 is a facultative anaerobe able to respire nitrate and nitric oxide. It has a broad metabolic range and a complete TCA cycle (similar to *S. alvi)*, and can assimilate many organic acids including acetate, one of the major metabolites produced in the worker gut microbiome^14,81^.

The shift in taxonomic representation following antibiotic treatment is also consistent with previous work determining the prevalence of antibiotic resistance genes in gram-negative bacteria including Alpha 2.1 (*Commensilibacter*), *S. alvi,* and Alpha 1 (*Bartonella apis)*^33^. When subject to tetracycline, Tian et al. found that ten of 13 tested strains of *G. apicola* were antibiotic resistant, while all tested isolates (n = 11) of *S. alvi* were resistant. Genomes of replacement bacteria in our study may contain antibiotic resistance genes, but their functional gene content concerning community dynamics or host niche is unknown. Further work is needed to demonstrate whether decreased microbiome diversity following antibiotic treatment significantly affects microbiome function. Within a colony, antibiotics are collected, stored and endure as a viable molecule in honey. The molecular structure necessary for antibiotic efficacy decays to its non-antibiotic constituent parts at 5–9 weeks^66^. From that same study, it was concluded that oxytetracycline has much greater persistence in honey than does tetracycline^66^.

Our sampling design cannot distinguish the microbiotas of older established bees from those of newly emerged bees. While the guts of newly emerged bees may fail to establish antibiotic susceptible species, the microbiomes of older bees may be more resistant to antibiotic treatment due to a fully established biofilm structure. The bimodality of microbiome size associated with time one may be influenced by worker emergence time, with the youngest newly emerged bees assembling their adult microbiomes under the most concentrated antibiotic conditions. This is an important consideration when interpreting the data because we predict that an antibiotic would have a greater impact on newly emerged adult bees early in the process of microbiome transmission and assembly. This may in part explain the pattern of microbial abundance at time one, a period designed to sample the full extent of antibiotic induced dysbiosis. Although unrealistic in the large field setting, future work would benefit by marking age cohorts so field results can be calibrated by worker emergence time.

### Supplementary Information

Supplementary Figure S1.

Supplementary Table S1.

Supplementary Table S2.

Supplementary Table S3.

Supplementary Table S4.

Supplementary Table S5.

## Data Availability

The raw sequence read datasets generated for this study are deposited in GenBank, Sequence Read Archive PRJNA957429.
