# Supplementary figures and images for: A longitudinal field study of commercial honey bees shows that non-native probiotics do not rescue antibiotic treatment, and are generally not beneficial
Source: Sci Rep. 2024 Jan 23;14:1954. doi: 10.1038/s41598-024-52118-z (PMC10806037; doi:10.1038/s41598-024-52118-z)

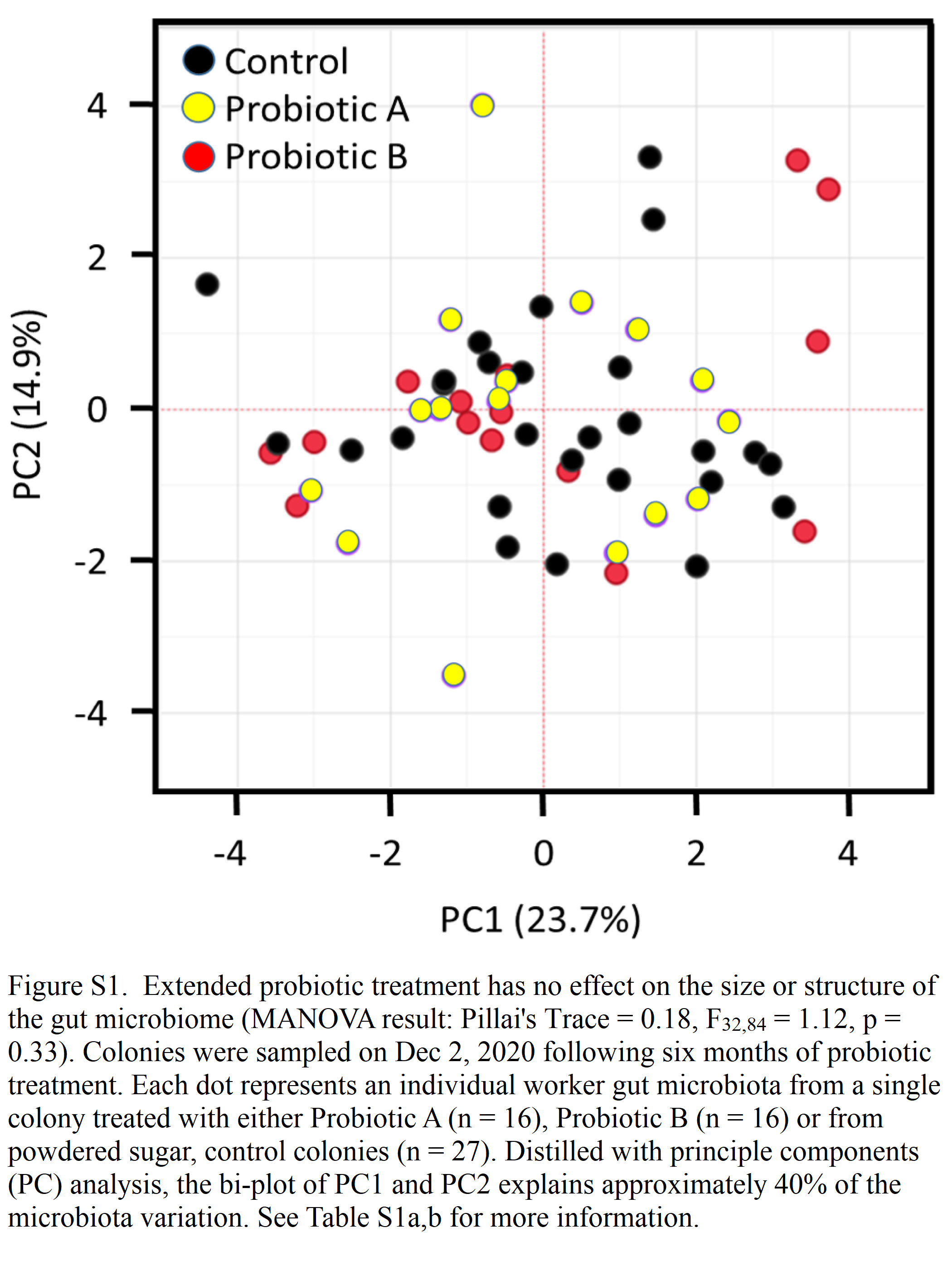

Supplement: Supplementary file 1 — Supplementary Figure S1. [file 41598_2024_52118_MOESM1_ESM.tif]
